# Tobacco use in Crohn's disease patients and association with disease outcomes in the United States Medicaid population, 2010–2019

**DOI:** 10.1002/jgh3.12893

**Published:** 2023-03-31

**Authors:** Ryan A. Jasper, Po‐Hung Chen, Reeha Patel, Shelly Joseph, Steven D. Miller, Susan Hutfless

**Affiliations:** ^1^ Whiting School of Engineering Johns Hopkins University Baltimore Maryland USA; ^2^ Division of Gastroenterology and Hepatology, Department of Medicine Johns Hopkins University School of Medicine Baltimore Maryland USA; ^3^ Krieger School of Arts and Sciences Johns Hopkins University Baltimore Maryland USA; ^4^ Division of Pediatric Gastroenterology, Hepatology, and Nutrition, Department of Pediatrics New York University School of Medicine New York New York USA; ^5^ Division of Pediatric Gastroenterology, Hepatology and Nutrition, Department of Pediatrics Johns Hopkins University School of Medicine Baltimore Maryland USA; ^6^ The Gastrointestinal Epidemiology Research Center Johns Hopkins University Baltimore Maryland USA

**Keywords:** Crohn's disease, epidemiology, Medicaid, smoking, tobacco

## Abstract

**Background and Aim:**

To identify demographic factors associated with tobacco use in Crohn's disease (CD) patients in the US Medicaid population and examine how tobacco use affects disease outcomes.

**Methods:**

We included Medicaid‐eligible patients who had ≥1 ICD code for CD, and 1 year of eligibility before and after the initial encounter. We used ICD codes to identify tobacco use with respect to the time of diagnosis and used logistic regression to identify the association between age, sex, and race with tobacco use at any point before diagnosis and after diagnosis, and determine the association of tobacco use before and after diagnosis on disease outcomes.

**Results:**

We identified 98 176 eligible patients; 74.5% had no documented use of tobacco and 25.5% used tobacco at some point; 21.1% had used tobacco before their CD diagnosis and 11.8% had used tobacco after diagnosis. The population that used tobacco had a higher proportion of women, those who were White, non‐Hispanic, and those in their middle ages (21–60) than the group that did not use tobacco. Tobacco use before diagnosis resulted in higher risk of hospitalization and surgery (OR: 1.85 and 1.36, respectively).

**Conclusion:**

Within the CD Medicaid population, tobacco use is more common in women than men, which differs from the general population, which is possibly a result of using diagnostic codes rather than survey data. Smoking cessation efforts should especially be directed at younger people who are at risk for CD, due to increased risk for more adverse outcomes among those who use tobacco before diagnosis.

## Graphical abstract

In the Medicaid Crohn's Disease Population, those aged 31–50, women, and those of non‐Hispanic White race have the highest tobacco use rate. We identified tobacco use status by before diagnosis and after diagnosis, and tobacco use before diagnosis results in worse disease outcomes, whereas tobacco use after diagnosis does not.

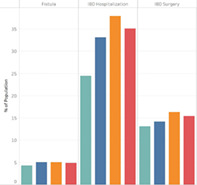

## Background

In 2018, 19.7% of adults in the United States reported using tobacco, according to the National Health Interview Survey (NHIS), which collects yearly health interview data from a selection of households in the United States.^1^ Since 1965, tobacco use rates have steadily decreased, both in men and women, as a result of ongoing efforts to promote smoking cessation.^2^ In the Medicaid population, the prevalence of tobacco use in 2017 was estimated at 28.2%, using data from the NHIS.^3^ By 2019, the NHIS reported that the prevalence of tobacco use in the Medicaid population increased to 30.0%.^4^

Crohn's disease (CD) is a chronic, primarily gastrointestinal, inflammatory disease that results from a combination of genetic and environmental factors.^5^ One known environmental factor that can increase the risk of CD is tobacco use.^5^ Meta‐analyses published in 1989 and 2006 both found significant associations between smoking and the risk of CD (OR 1.8 and 2.0, respectively).^6^, ^7^ Moreover, smoking is associated with an increased risk of surgery, need for immunosuppressive medications, and relapse.^8^, ^9^ The negative impact of smoking on CD outcomes is typically stronger in women than men.^9^, ^10^ However, these meta‐analyses relied primarily on studies that identified smoking status from self‐reported data through an interview or a questionnaire, whereas Medicaid has documented medical claims to identify tobacco use status. In addition, prior studies typically categorized smokers into current smokers, former smokers, and never smokers, but did not classify tobacco use status with respect to CD diagnosis because of their cross‐sectional nature.

We aimed to identify the demographic factors associated with tobacco use in CD patients in the US Medicaid population and examine how tobacco use affects various outcomes, including medication use, fistula, hospitalization, and surgery, using claims data between 2010 and 2019.

## Methods

### Medicaid cohort eligibility

Our study is a retrospective cohort study using administrative claims data from the national Medicaid databases. Medicaid is federally funded and administered by each state to provide health coverage to those who meet certain criteria. All states use income level as a criterion for eligibility, while some also include additional populations such as pregnant women, children, elderly people, and people with disabilities. The additional populations included vary on a state‐by‐state basis.^11^

### Crohn's disease case definition

We used the International Classification of Diseases, Ninth and Tenth Revision, Clinical Modification (ICD‐9‐CM and ICD‐10‐CM) codes to identify CD. For inclusion in our study, we defined CD as having at least one encounter with CD diagnostic codes (ICD‐9‐CM 555 and ICD‐10‐CM K50). We included all people aged 0–105 at the time of CD diagnosis and all encounters between 2010 and 2019. We also required that people in our study population had 1 year of eligibility in Medicaid prior to the initial CD diagnosis and 1 year of eligibility in Medicaid after CD diagnosis. We defined Medicaid eligibility as having 15 or more days of eligibility in a given month, to be considered eligible in that month. A person was no longer considered eligible if they had three consecutive months of ineligibility. We excluded those who had missing date of birth or sex and all encounters with missing dates.

### Tobacco use

We used the ICD‐9‐CM and ICD‐10‐CM codes to identify tobacco use. The ICD‐9 codes included were 3051 and V1582, and the ICD‐10 codes are Z87891, F17, T652, Z716, and Z720 (Table S1). Tobacco use was divided into three main categories. A diagnosis of tobacco use before the first recorded CD diagnosis date was classified as “tobacco before CD”; a diagnosis of tobacco use on or after CD diagnosis was classified as “tobacco after CD”; and a diagnosis of tobacco use at any point during the study period was classified as “ever having used tobacco”.

### Race/ethnicity

We used the race and ethnicity groups provided by TAF files to classify study participants into one of the following categories: White non‐Hispanic, Black non‐Hispanic, and other/unknown.^12^ The records identified using the MAX database were converted to the TAF definitions.^13^

### Outcomes

We examined outcomes related to timing of tobacco use documentation as well as clinical outcomes significant to CD.

### Medications

For our study population, we considered all prescriptions during the study period for many inflammatory bowel disease (IBD)‐related medications. We categorized these medications into their respective classes, namely biologics, tumor necrosis factor (TNF) inhibitors, immunomodulators, steroids, and antibiotics (Table S1).

### Hospitalization

We included inpatient hospitalizations following CD diagnosis as well as those that were associated with IBD, to define an IBD‐related hospitalization in our study population (Table S1).

### Surgery

We used the ICD‐9‐CM and ICD‐10‐CM procedure codes to identify IBD‐related surgeries in our study population. Such surgeries include closure, excision, or the repair of a fistula, intestinal excision or resection, ostomy formation, and abscess drainage (Table S1).

### Fistula

We used ICD‐9‐CM and ICD‐10‐CM diagnostic codes to identify instances of fistulas in our study population. We included any fistulas that occurred at or after the CD diagnosis. Each fistula was categorized as an intestinal fistula, a perianal fistula, or a rectovaginal fistula (Table S1).

### Statistics

We calculated the prevalence of tobacco use among people newly diagnosed with CD during the study period. We performed multivariable logistic regression to analyze factors associated with tobacco use in CD patients, including age, sex, race, and the type of Medicaid eligibility. We also used logistic regression to examine factors associated with hospitalization, surgery, medication use, and fistula, including tobacco use before diagnosis and tobacco use after diagnosis. All analyses were performed in SAS Enterprise Guide. The code related to this project is publicly available.

### Sensitivity analysis

Many women become eligible for Medicaid when they are pregnant; tobacco use status is commonly recorded for pregnant women. We aimed to examine whether the association differed between men and women. We conducted a sensitivity analysis by stratifying our population by sex and examining the association between tobacco use before and after diagnosis on CD outcomes.

## Results

Of the 98 176 Medicaid beneficiaries who had one or more CD encounters, at least 365 days of eligibility before their CD diagnosis, and at least 1 year follow‐up, 73 187 (74.5%) never used tobacco, 24 989 (25.5%) used tobacco at any point, 20 734 (21.1%) used tobacco before their CD diagnosis, and 11 591 (11.8%) used tobacco on or after their CD diagnosis. Many characteristics of those who never used tobacco and those who used tobacco differed (Table 1).

**Table 1:** Demographics of the Medicaid population with 1+ Crohn's disease (CD) diagnosis code and 365 days eligibility before and after diagnosis, 2010–2019

		Never used tobacco	Used tobacco after CD diagnosis	Used tobacco before CD diagnosis	Ever used tobacco
*N*		73 187	11 591	20 734	24 989
Age at first Medicaid eligibility (%)	0–5	0.66	0.00	0.00	0.00
	6–10	5.02	0.1	0.07	0.08
	11–20	15.18	6.88	4.72	5.63
	21–30	14.19	21.23	18.97	19.25
	31–40	15.02	23.29	21.16	21.38
	41–50	16.35	24.8	25.06	24.55
	51–60	15.89	17.73	20.21	19.74
	61–70	10.34	4.88	7.38	7.1
	71–105	7.33	1.07	2.43	2.27
Race (%)	White non‐Hispanic	51.39	69.85	66.26	66.43
	Black non‐Hispanic	14.45	15.41	16.54	16.38
	Other/unknown	34.16	14.74	17.2	17.19
Sex (%)	Female	61.96	69.58	66.44	66.75
First year of Medicaid eligibility (%)	2010	60.49	88.31	75.49	77.96
	2011	5.03	6.72	7.24	6.9
	2012	3.17	2.68	3.63	3.42
	2013	2.37	0.94	1.99	1.83
	2014	7.11	1.35	5.4	4.71
	2015	4.31	0.00	1.37	1.14
	2016	16.05	0.00	4.87	4.04
	2017	1.46	0.00	0.00	0.00
First year of eligible CD encounter (%)	2011	16.83	37.75	21.43	25.64
	2012	15.44	30.31	21.81	22.99
	2013	11.78	18.72	15.67	15.64
	2014	7.65	8.64	9.33	8.85
	2015	8.13	4.57	9.41	8.34
	2016	8.71	0.00	7.04	5.84
	2017	16.76	0.00	8.6	7.14
	2018	14.69	0.00	6.7	5.56
Age at first CD encounter (%)	11–20	17.99	4.83	2.47	3.43
	21–30	13.51	18.38	14.93	15.54
	31–40	14.84	24.16	21.88	22.13
	41–50	15.3	23.52	22.74	22.51
	51–60	17.81	21.73	24.98	24.1
	61–70	11.17	5.81	9.43	8.98
	71–105	9.38	1.57	3.57	3.31
Place of service at first CD encounter (%)	Office	32.87	26.83	22.34	24.42
	Inpatient hospital	20.5	23.75	32.29	29.18
	Outpatient hospital	18.76	26.53	21.28	22.33
	Emergency room/hospital	7.32	11.6	11.22	10.97
	Other	20.56	11.28	12.87	13.09
1+ CD code (%)		100	100	100	100
2+ CD codes (%)		51.72	59.41	53.39	54.62
Tobacco (%)		0.00	100	100	100
Steroid (%)		54.19	66.65	61.23	61.59
Antibiotic (%)		50.3	64.48	58.99	59.43
5‐ASA (%)		17.19	16.12	13.87	14.39
Immunomodulator (%)		9.4	6.72	5.83	6.15
6‐Mercaptopurine (%)		3.31	2.24	1.8	1.98
Azathioprine (%)		3.62	2.95	2.7	2.78
Methotrexate (%)		3.26	1.99	1.77	1.82
Biologic (%)		12.35	8.96	8.02	8.39
Anti‐TNF (%)		11.39	8.73	7.62	8.01
Adalimumab (%)		5.97	5.31	4.63	4.81
Certolizumab pegol (%)		0.5	0.77	0.52	0.59
Golimumab (%)		0.14	0.00	0.1	0.1
Infliximab (%)		6.43	4.31	3.72	3.96
JAK inhibitor (%)		0.17	0.00	0.07	0.06
Tofacitinib (%)		0.17	0.00	0.07	0.06
Upadacitinib (%)		0.00	0.00	0.00	0.00
Natalizumab (%)		0.04	0.00	0.00	0.00
Ustekinumab (%)		0.9	0.43	0.51	0.48
Vedolizumab (%)		1.32	0.64	0.7	0.7
Cyclosporine (%)		0.02	0.00	0.00	0.00
TPN (%)		6.82	6.25	8.89	8.17
Fistula (%)		4.31	5.05	5.07	4.92
Intestinal fistula (%)		1.96	2.67	2.83	2.67
Perianal fistula (%)		2.5	2.68	2.6	2.57
Rectovaginal fistula (%)		0.46	0.86	0.74	0.73
IBD hospitalization (%)		24.46	33.12	37.92	35.11
IBD surgery (%)		13.14	14.21	16.36	15.45
No. of CD encounters (mean)		8.42	13.2	9.85	10.35
Age at first CD encounter (mean)		43.05	41.8	45.15	44.46
Age at first Medicaid eligibility (mean)		40.38	39.93	42.41	41.9
No. of eligible days 2010–2019 (mean)		2458.91	2695.93	2648.88	2659.67

ASA, aminosalicylate treatment; CD, Crohn's disease; IBD, inflammatory bowel disease; anti‐TNF, anti‐tumor necrosis factor‐α treatment.

### Factors associated with ever tobacco use

**Figure 1:**
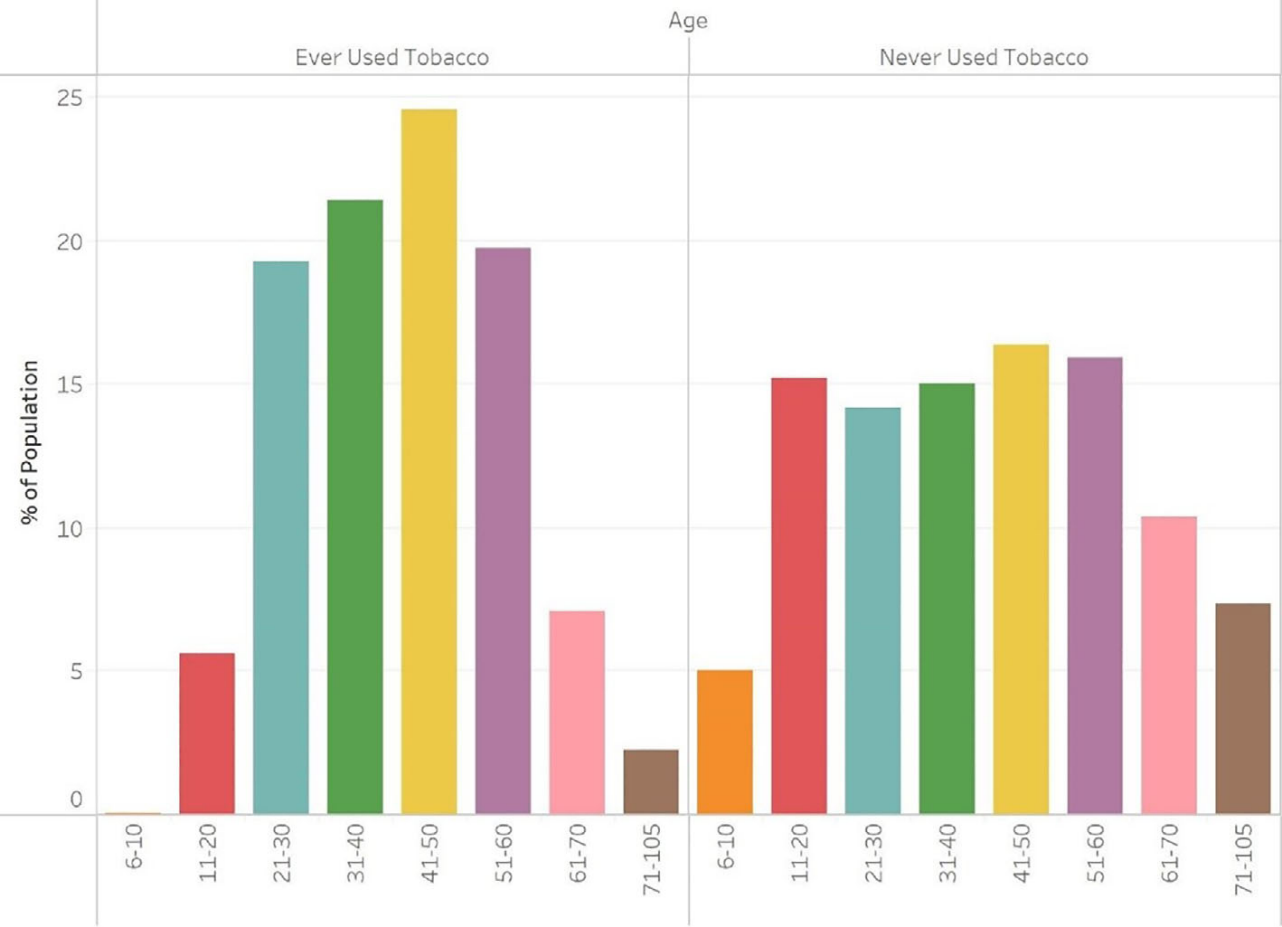
Age Distribution of tobacco users and non‐tobacco users in Medicaid Crohn's disease population, 2010–2019.

**Figure 2:**
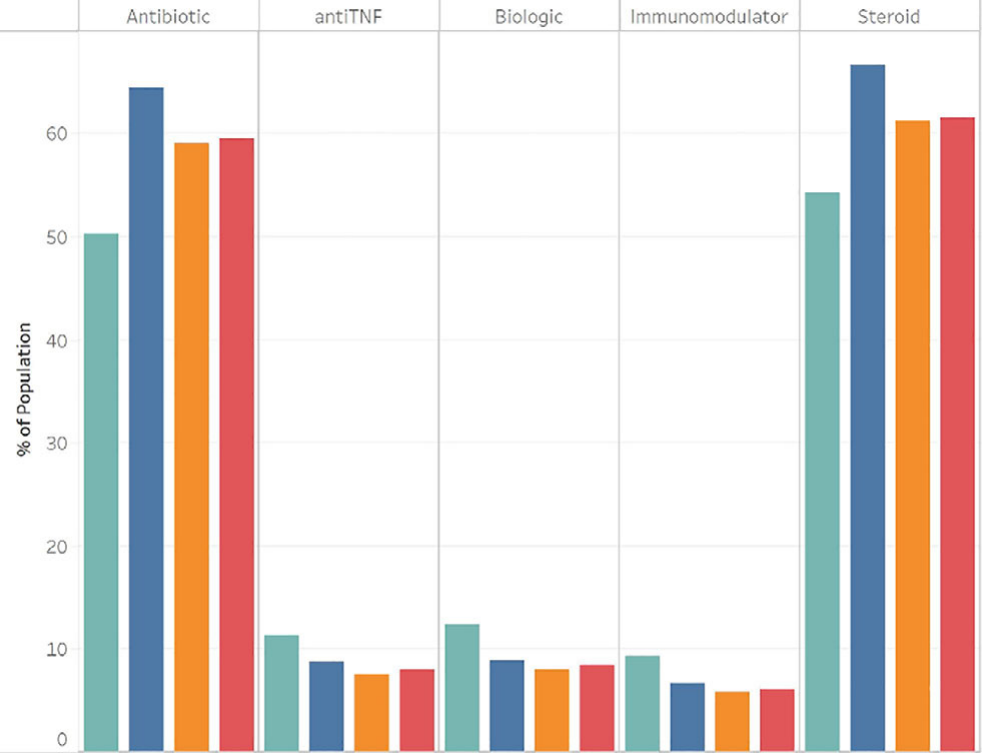
Medication type use in Medicaid Crohn's disease patients by tobacco use status, 2010–2019. Tobacco use status: 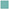, Never used tobacco; 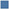, Used tobacco after CD dx; 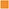, Used tobacco before CD dx; 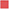, Ever used tobacco.

**Figure 3:**
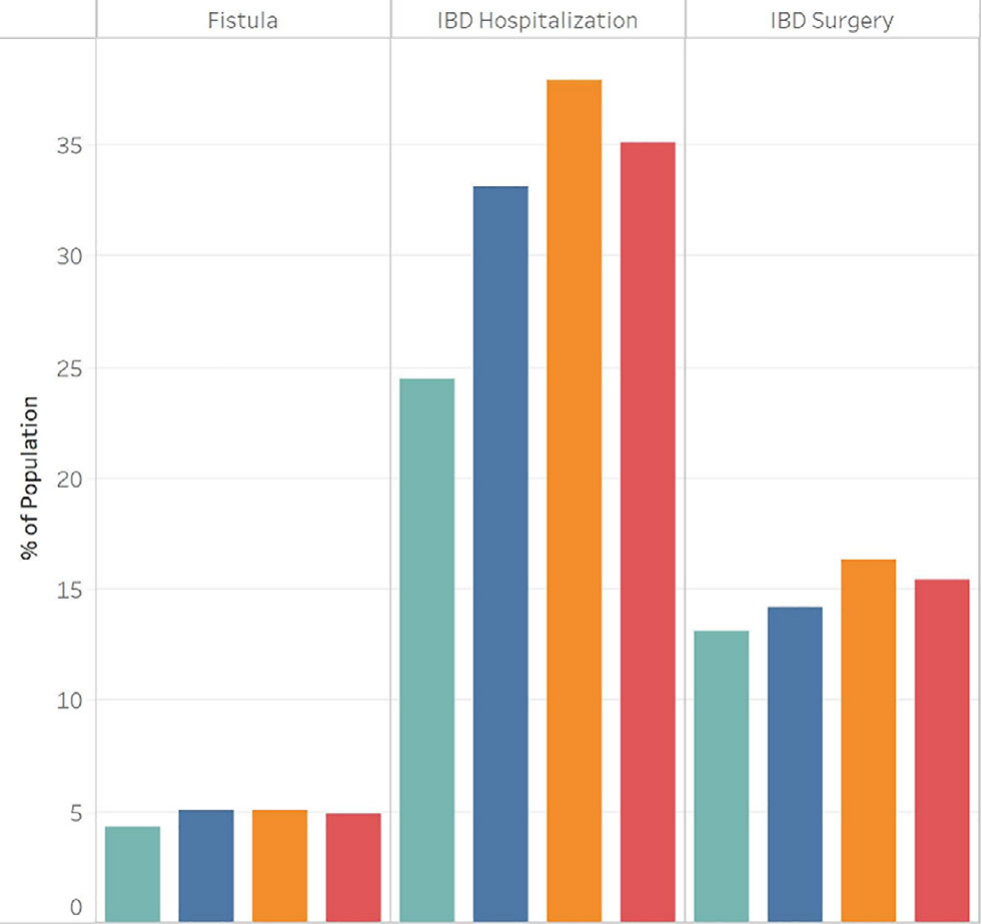
Crohn's disease outcomes in Medicaid population by tobacco status, 2010–2019. Tobacco use status: 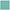, Never used tobacco; 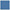, Used tobacco after CD dx; 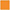, Used tobacco before CD dx; 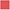, Ever used tobacco.

Tobacco users were more likely than non‐tobacco users to be White non‐Hispanic (66.4% vs 51.4%) or Black non‐Hispanic (16.4% vs 14.5%), but less likely to be of other or unknown race (17.2% vs 34.2%). The Black non‐Hispanic population was less likely to use tobacco than the White non‐Hispanic population (odds ratio [OR] 0.86, 95% confidence interval [CI]: 0.82–0.89), while those of unknown race were significantly less likely than those of White race to use tobacco (OR 0.46, 95% CI: 0.44–0.47) (Table 2). We found that those who used tobacco were more likely to be female than those who did not (66.8% vs 62.0%, OR 1.08, 95% CI: 1.05–1.12). The population of those who never used tobacco had higher rates of people in the youngest (0–5, 6–10, 11–20) and oldest (61–70, 71–105) age groups, whereas tobacco users were more likely to be in the middle (21–60) age range (Fig. 1). The age groups of 31–40, 41–50, and 51–60 were all more likely than those aged 21–30 to use tobacco at any point (OR 1.27, 1.27, and 1.15, respectively). Those aged 11–20, 61–70, and 71–105 were all less likely than those aged 21–30 to use tobacco at any point (OR 0.16, 0.71, and 0.32, respectively). We observed that those who never used tobacco were less likely than tobacco users to use antibiotics and steroids, but more likely to use biologics, immunomodulators, and TNF inhibitors (Fig. 2). IBD hospitalization rates, IBD surgery rates, and fistula rates of all three types were lower in the non‐tobacco use population (Fig. 3).

**Table 2:** Odds ratios for effect of age, race, and sex on tobacco use in Medicaid Crohn's disease (CD) population, 2010–2019

	Ever used tobacco			Used tobacco after CD dx			Used tobacco before CD dx		
	Odds ratio	Lower CL^†^	Upper CL^†^	Odds ratio	Lower CL^†^	Upper CL^†^	Odds ratio	Lower CL	Upper CL
Age 11–20	0.162	0.149	0.176	0.358	0.323	0.398	0.133	0.120	0.147
Age 21–30 Reference									
Age 31–40	1.269	1.206	1.335	0.976	0.911	1.044	1.291	1.224	1.363
Age 41–50	1.270	1.207	1.336	0.918	0.857	0.983	1.329	1.259	1.402
Age 51–60	1.154	1.098	1.213	0.699	0.652	0.750	1.263	1.198	1.331
Age 61–70	0.706	0.664	0.751	0.350	0.318	0.387	0.822	0.770	0.877
Age 71–105	0.317	0.292	0.345	0.153	0.130	0.179	0.396	0.362	0.432
White non‐Hispanic Reference									
Black non‐Hispanic	0.855	0.820	0.893	0.774	0.729	0.822	0.888	0.849	0.928
Other/unknown	0.456	0.438	0.474	0.559	0.527	0.593	0.473	0.454	0.493
Female	1.081	1.047	1.117	1.214	1.159	1.272	1.046	1.011	1.082

^†^ Confidence level is 95%.

CD, Crohn's disease; dx, diagnosis.

### Factors associated with tobacco use before versus after CD diagnosis

The trend of women being more likely to use tobacco than men was consistent when considering tobacco use before CD diagnosis (OR 1.05; 95% CI: 1.01–1.08) and after CD diagnosis (OR 1.21; 95% CI: 1.16–1.27) (Table 2). In addition, those who were identified as non‐Hispanic Black were less likely to use tobacco than those who were non‐Hispanic White before and after CD diagnosis (OR 0.89 and OR 0.77, respectively). Those of other or unknown race were also significantly less likely than those of White non‐Hispanic race to use tobacco before and after CD diagnosis (OR 0.47 and 0.56). The 31–40, 41–50, and 51–60 age groups were also more likely than those aged 21–30 to use tobacco before CD diagnosis (OR 1.29, 1.33, 1.26, respectively). However, those aged 41–50 and 51–60 were less likely than the 21–30 group to use tobacco after CD diagnosis (OR 0.92 and 0.70). The 31–40 age group had a similar likelihood of tobacco use after CD diagnosis as the 21–30 age group (OR 0.98; 95% CI: 0.91–1.04).

### Factors associated with medication, hospitalization, and surgery outcomes

Within the population that used tobacco, those who used tobacco after CD diagnosis had higher rates of medication use for all medication classes examined, compared to those who used tobacco before CD diagnosis (Fig. 2). However, those who used tobacco before CD diagnosis had higher rates of hospitalization, surgery, and fistula than those who used tobacco on or after diagnosis (Fig. 3).

Those who used tobacco before CD diagnosis were less likely to use TNF inhibitors and other biologics (OR 0.81 and 0.78), but more likely to use steroids (OR 1.23; 95% CI: 1.19–1.28) than those who did not use tobacco before CD diagnosis (Table 3). Tobacco use before CD diagnosis resulted in a significantly increased risk of an IBD‐related hospitalization (OR 1.85; 95% CI: 1.78–1.91), IBD‐related surgery (OR 1.36; 95% CI: 1.30–1.42), and fistula (OR 1.15; 95% CI: 1.06–1.25).

**Table 3:** Odds ratios for effect of tobacco use on Crohn's disease (CD) outcomes in Medicaid population, 2010–2019

	Used tobacco after CD dx			Used tobacco before CD dx		
	Odds ratio	Lower CL†	Upper CL	Odds ratio	Lower CL†	Upper CL†
Anti‐TNF	0.909	0.843	0.981	0.809	0.759	0.862
Biologic	0.865	0.803	0.931	0.781	0.734	0.830
Fistula	1.027	0.931	1.133	1.150	1.062	1.245
IBD hospitalization	0.980	0.936	1.027	1.846	1.780	1.914
IBD surgery	0.932	0.877	0.991	1.357	1.295	1.423
Steroid	1.442	1.378	1.509	1.230	1.187	1.275

^†^ Confidence level is 95%.

anti‐TNF, anti‐tumor necrosis factor‐α treatment; CD, Crohn's disease; dx, diagnosis; IBD, inflammatory bowel disease.

We found that those who used tobacco on or after CD diagnosis were also less likely to use TNF inhibitors and other biologics (OR 0.91 and 0.87) but more likely to use steroids (OR 1.44; 95% CI: 1.38–1.51). However, tobacco use after CD diagnosis did not increase the risk of hospitalization (OR 0.98; 95% CI: 0.94–1.03) or fistula (OR 1.03; 95% CI: 0.93–1.33) and slightly decreased risk of surgery (OR 0.93; 95% CI: 0.88–0.99).

### Sensitivity analysis

In the sensitivity analysis, stratified by sex, we observed that there were no significant differences in the direction or magnitude of the ORs considering the association between tobacco use before and after diagnosis and use of anti‐TNF drugs, biologics, steroids, hospitalization, surgery, and fistula in men compared to women (Tables S2, 3a,b).

## Discussion

We found that in the CD population, women, those of White non‐Hispanic race, and those aged 31–40 and 41–50 were most likely to use tobacco at any point. Those who used tobacco were less likely to use certain CD medications but more likely to use others. Using tobacco before diagnosis increased the risk of hospitalization, surgery, and fistula, although using tobacco after diagnosis did not increase that risk. Tobacco users were at a lower risk for using TNF inhibitors and biologics but at a higher risk for the use of steroids.

The prevalence of tobacco use in the CD Medicaid population was higher than the rate of tobacco use in the general population, which is consistent with the NHIS estimates of the Medicaid tobacco use rate.^1^, ^4^ However, our estimates were slightly lower than the NHIS estimates of the Medicaid population, which may have resulted from our use of codes to identify tobacco use, causing an underestimation.^14^

Our finding that, among the Medicaid CD population, women are at a higher risk for tobacco use than men is consistent with a 2015 cohort study in Switzerland, which found within a CD population 42.8% of women and 35.8% of men smoked.^15^ However, a more recent cohort study in Taiwan found that in the CD group, those who smoked were more likely to be men than those who did not.^16^ In addition, the NHIS survey conducted in the general US population has found a higher prevalence of tobacco use in men than women.^2^, ^4^ One possible explanation for this difference is that this data included participants representative of the entire United States and not the CD population. Additionally, this discrepancy could be due to the increased number of pregnant women who are eligible for Medicaid, and therefore represents an increased proportion of the Medicaid population, who are more likely to be asked about their tobacco use status.

The observed increased risk of tobacco use in White non‐Hispanic patients compared to Black non‐Hispanic patients is consistent with the NHIS data, which found that in 2019, 23.3% of White non‐Hispanic participants reported using tobacco compared to 20.7% of Black non‐Hispanic participants reporting using tobacco.^4^

Our finding that tobacco users, regardless of its use before or after diagnosis, are less likely to use TNF inhibitors and other biologics may be explained by studies that have found increased antibodies in response to anti‐TNF drugs in tobacco users and decreased chance of remission compared to non‐tobacco users when using another biologic, vedolizumab.^17^ Despite possible increased antibodies, it was reported that there was no significant difference in response to the anti‐TNF infliximab between tobacco users and non‐tobacco users in the CD population.^18^ The observed increased risk of steroid use in tobacco users is consistent with previous studies and is likely a result of increased risk of disease flares as a result of tobacco use.^8^, ^17^, ^19^ Although the reduced rates of biologic prescription may be a direct effect of publicity around reduced efficacy among CD patients who use tobacco, it is also possible that tobacco use represents a hidden variable, such as living in a site with lower access to gastroenterology specialty care, which may result in lower use of biologic medications.

Previous systematic reviews on the effect of tobacco use on the risk of surgery in IBD patients found that tobacco users were at a higher risk for surgery than non‐tobacco users, but those who previously used tobacco and quit were not at an increased risk compared to those who never used tobacco.^19^, ^20^ Similarly, previous studies have shown that tobacco users have on average more hospitalizations than non‐tobacco users.^21^ Our results suggest that tobacco use before CD diagnosis results in increased risks of hospitalization and surgery, while tobacco use after CD diagnosis is less consequential towards these outcomes. The reduced usage of biologic medications among patients with CD who smoke may also partly explain the higher likelihood of progression to surgery among this population. Although previous studies have indicated that these negative effects of tobacco are much stronger in women than men with CD, our sensitivity analysis found minimal differences between the two in all outcomes that we considered.^9^, ^10^

There were several factors in the study design that created limitations. First, we identified tobacco use using ICD codes, which have been found to underestimate tobacco use when compared to direct interviewing methods, although the difference was not statistically significant.^14^ As a result, there were likely instances of true tobacco users being classified as non‐tobacco users, leading to an underestimation of tobacco use in our study population. Because of our use of ICD codes, we were unable to determine whether tobacco users would be classified as former tobacco users or current tobacco users, but were able to discern tobacco use status with respect to the time of CD diagnosis. In addition, there may be a discrepancy in the demographics of people more likely to be asked about their tobacco use status, such as pregnant women, which could skew the observed differences in tobacco use rates by sex. Although we elected to only require 1 CD code to be eligible for our study to avoid excluding CD patients, this case definition does yield inconsistent accuracy in the diagnosis.^22^ As a result, there might have been participants included in our study who did not actually have CD.

## Conclusion

This study helps to inform the need for further efforts to promote smoking cessation in the general population, with emphasized efforts towards younger people, since smoking before diagnosis is linked to worse outcomes, including higher risks of hospitalization, surgery, and fistula. Further investigation into the mechanism by which tobacco use before CD diagnosis is particularly harmful to outcomes when compared to tobacco use after diagnosis is needed. In addition, further inquiry is needed to better understand how and why tobacco impacts the effectiveness of medications commonly used in a CD treatment regimen.

## Ethics statement

The Johns Hopkins Institutional Review Board reviewed and approved the study protocol (IRB00206614).

## Supporting information


**Data S1.** Supporting Information.

## Data Availability

Codes used to identify the cohort, medications, hospitalization, surgery, smoking, and other characteristics of the Crohn's disease and general Medicaid population are available in Supplemental files. SAS code is available in a public GitHub repository (https://github.com/susanmhutfless/playground/tree/master/crohns/code/sas/Advanced).
