# Supplementary material for: Tobacco use in Crohn's disease patients and association with disease outcomes in the United States Medicaid population, 2010–2019
Source: JGH Open. 2023 Mar 31;7(4):291–8. doi: 10.1002/jgh3.12893 (PMC10134760; doi:10.1002/jgh3.12893)
Supplement: Supplementary file 1 — Data S1. Supporting Information. [file JGH3-7-291-s001.docx]

Supplement.

Supplemental Table 1: Codes used to identify eligibility group, race, tobacco use, medications, fistula, hospitalizations, and surgical procedures

Code Available at https://github.com/susanmhutfless/playground/tree/master/crohns/code/sas/Advanced

| **Category** | **Case definition/codes used** |
| --- | --- |
| Eligibility group | if start_yr<2015 then do;  if el_max_elgblty_cd_ltst in('11','14','16','17','24','31','34','35') then do; elig_income=1; elig_grp=1;end;  if el_max_elgblty_cd_ltst in('12','15','22','32','42','52') then do; elig_dsblty=1;  elig_grp=2;end;  if elig_income=. and elig_dsblty=. then do; elig_other=1;  elig_grp=3; end;  end;    if start_yr>=2015 then do;  if el_max_elgblty_cd_ltst in('04','05','06','07','08','09','11','13','17','18','25','28','29','30','31','33','35','37','38','47','48','49','61','62','63','64','65','67','68','70','72','73','74','75')  then do; elig_income=1; elig_grp=1;end;  if el_max_elgblty_cd_ltst in('12','15','16','19','20','21','22','23','24','39','42','45','50','51','52','53','54','55','56','59','60','69')  then do; elig_dsblty=1;  elig_grp=2;end;  if elig_income=. and elig_dsblty=. then do; elig_other=1;  elig_grp=3; end;  end; |
| Race | if start_yr<2015 then do;  if den_race_eth_cd in(0,9,.) then den_race_eth_cd=9;  if den_race_eth_cd=3 then do; new_den_race_eth_cd=4; den_race_eth_cd=.; end;  if den_race_eth_cd=4 then do; new_den_race_eth_cd=3; den_race_eth_cd=.; end;  if den_race_eth_cd=6 then do; new_den_race_eth_cd=5; den_race_eth_cd=.; end;  if den_race_eth_cd=8 then den_race_eth_cd=6;  if den_race_eth_cd in(5,7) then den_race_eth_cd=7;    if start_yr>=2015 then do;  if den_race_eth_cd in(0,8,9,.) then den_race_eth_cd=9; |
|  |  |
| Tobacco | Based on diagnosis codes that are complete match or substring3 or substring4    /** substance - tobacco **/  %let  tobacco_diag_criteria   = '3051' 'V1582' 'Z87891';  %let  tobacco_diag3_criteria   = 'F17';  %let  tobacco_diag4_criteria   = 'T652' 'Z716' 'Z720';   \| Source \| Code \| Description \| \| --- \| --- \| --- \| \| ICD-9 \| 3051 \| Tobacco use disorder \| \|  \| V1582 \| Personal history of tobacco use \| \| ICD-10 \| Z87891 \| Personal history of nicotine dependence \| \|  \| F17 \| Nicotine dependence \| \|  \| T652 \| Toxic effect of tobacco and nicotine \| \|  \| Z716 \| Tobacco abuse counseling \| \|  \| Z720 \| Tobacco use \| |
| Corticosteroids | if pharm_classes = "CORTICOSTEROID [EPC],CORTICOSTEROID HORMONE RECEPTOR AGONISTS [MOA]"                             then drug_steroids=1;  %let hcpcs_steroid_criteria = 'J1020' 'J1030' 'J1040' 'J2920' 'J2930' ;*NOT INCLUDING ORAL J7509, J7506, J7512;*Not including G codes or poisoning |
| Antibiotic | where pharm_classes contains "ANTIBACTERIAL";           drug_antibiotics=1; |
| Aminosalicylate | if pharm_classes = "AMINOSALICYLATE [EPC],AMINOSALICYLIC ACIDS [CS]"                             then drug_5asa=1; |
| Immunomodulator | Azathioprine, Methotrexate, Mercaptopurine |
| Six-mercaptopurine | if NONPROPRIETARYNAME = 'MERCAPTOPURINE' then do;                             drug_6mp=1;                             drug_immunomodulator=1; |
| Azathioprine | if substr(NONPROPRIETARYNAME,1,12) = 'AZATHIOPRINE' then do;                             drug_AZA=1;                             drug_immunomodulator=1;  %let hcpcs_aza_criteria          = 'J7500' 'J7501' |
| Methotrexate | if substr(NONPROPRIETARYNAME,1,12) = 'METHOTREXATE' then do;                             drug_MTX=1;                             drug_immunomodulator=1;    %let hcpcs_mtx_criteria          = 'J8610' 'J9250' 'J9260' |
| Biologic | Adalimumab, Certolizumab, Golimumab, Infliximab, Tofacitinib, Upadacitinib, Natalizumab, Ustekinumab, Vedolizumab, Ozanimod |
| Anti-TNF | Adalimumab, Certolizumab, Golimumab, Infliximab |
| Adalimumab | if NONPROPRIETARYNAME in:( 'ADALIMUMAB') then do;                             drug_ada=1;                             drug_antiTNF=1;                             drug_biologic=1;    %let hcpcs_ada_criteria     = 'J0135' |
| Certolizumab pegol | if NONPROPRIETARYNAME in:( 'CERTOLIZUMAB') then do;                             drug_cert=1;                             drug_antiTNF=1;                             drug_biologic=1;    %let hcpcs_cert_peg_criteria= 'J0717' |
| Golimumab | if NONPROPRIETARYNAME in:( 'GOLIMUMAB') then do;                             drug_golim=1;                             drug_antiTNF=1;                             drug_biologic=1;    %let hcpcs_golim_criteria  = 'J1602' |
| Infliximab | if NONPROPRIETARYNAME in:('INFLIXIMAB') then do;                             drug_inflix=1;                             drug_antiTNF=1;                             drug_biologic=1;    %let hcpcs_inflix_criteria  = 'J1745' 'Q5102' 'Q5103' 'Q5104' 'Q5121' |
| JAK inhibitor | Tofacitinib, Upadacitinib |
| Tofacitinib | if NONPROPRIETARYNAME in:( 'TOFACITINIB') then do;                             drug_tofa=1;                             drug_biologic=1;                             drug_jak=1; |
| Upadacitinib | if NONPROPRIETARYNAME in:( 'UPADACITINIB') then do;                             drug_upa=1;                             drug_biologic=1;                             drug_jak=1; |
| Natalizumab | if NONPROPRIETARYNAME in:( 'NATALIZUMAB') then do;                             drug_natal=1;                             drug_biologic=1;    %let hcpcs_natal_criteria   = 'J2323' |
| Ustekinumab | if NONPROPRIETARYNAME in:( 'USTEKINUMAB') then do;                             drug_ust=1;                             drug_biologic=1;    %let hcpcs_ustek_criteria   = 'J3357' 'J3358' |
| Vedolizumab | if NONPROPRIETARYNAME in:( 'VEDOLIZUMAB') then do;                             drug_vedo=1;                             drug_biologic=1;    %let hcpcs_vedo_criteria    = 'J3380' |
| Cyclosporine | %let hcpcs_cyclo_criteria  = 'J7502' 'J7515' 'J7516' |
| Ozanimod | if NONPROPRIETARYNAME in:( 'OZANIMOD') then do;                             drug_oza=**1**;                             drug_biologic=**1**; |
| TPN | if pharm_classes in(                   "LIPID EMULSION [EPC],LIPIDS [CS]"                   "AMINO ACID [EPC],AMINO ACIDS [CS]"                   "LIPID EMULSION [EPC],LIPIDS [CS]"                   )             then tpn=**1**;             if PROPRIETARYNAME = 'CLINIMIX E'             then tpn=**1**;    %let  tpn_09_criteria_4   = '9915'                                ;    %let  tpn_10_criteria   = '3E0336Z' '3E0436Z' '3E0536Z' '3E0636Z';    %let  tpn_cpt_criteria =    'B4220' 'B4222' 'B4224' 'B4164' 'B4168'                                         'B4172' 'B4176' 'B4178' 'B4180' 'B4185'                                         'B4187' 'B4189' 'B4193' 'B4197' 'B4199'                                         'B4216' 'B4220' 'B4222' 'B4224' 'B5000'                                         'B5100' 'B5200' 'B9004' 'B9006'            ; |
| Fistula | ICD-9 Codes   \| 569.81 \| Fistula of intestine, excluding rectum and anus \| \| --- \| --- \| \| 596.1 \| Intestinovesical fistula \| \| 537.4 \| Fistula of stomach or duodenum \| \| 565.1 \| Anal fistula \| \| 619.1 \| Digestive-genital tract fistula, female \|     ICD-10 Codes   \| K63.2 \| Fistula of intestine \| \| --- \| --- \| \| N32.1 \| Vesicointestinal fistula \| \| K31.6 \| Fistula of stomach and duodenum \| \| K60.3 \| Anal fistula \| \| K60.4 \| Rectal fistula \| \| K60.5 \| Anorectal fistula \| \| K61.0 \| Anal abscess \| \| K61.1 \| Rectal abscess \| \| K61.3 \| Ischiorectal abscess \| \| K61.4 \| Intrasphincteric abscess \| \| K61.5 \| Supralevator abscess \| \| N82.2 \| Fistula of vagina to small intestine \| \| N82.3 \| Fistula of vagina to large intestine \| \| N82.4 \| Other female intestinal-genital tract fistulae \| |
| IBD hospitalization | Substring 3 of 555,556,K50,K51 included in diagnosis positions 1-25 on an inpatient claim   \| Source \| Code \| \| --- \| --- \| \| ICD-9 \| 555.x: Regional Enteritis \| \| ICD-9 \| 556.x: Ulcerative enterocolitis \| \| ICD-10 \| K50.xx: Crohn's disease [regional enteritis] \| \| ICD-10 \| K51.xx Ulcerative colitis \| |
| IBD surgery | ICD procedure and CPT codes used  ICD-9-CM uses substrings 2-4, ICD-10-CM uses substring 2 and additional criteria, CPT are exact match  %let  ibd_sur_09_criteria_2   = '46' ;  %let  ibd_sur_09_criteria_3   = '173' '456' '457' '458' '459' '484' '485' '486' '491' ;  %let  ibd_sur_09_criteria_4   = '4973' '5783' '7072' '7073' '7074' ;  %let  ibd_sur_10_criteria_2   = '0D';  ICD-10 PROCEDURE  if (substr(icd9_pr,1,2)) in ('0D') and     (substr(icd9_pr,3,1)) in ('B' 'P' 'Q' 'T') and     (substr(icd9_pr,4,1)) in ('8' '9' 'A' 'B' 'C' 'D' 'E' 'F' 'G' 'H' 'K' 'L' 'M' 'N' 'P')  %let  ibd_sur_cpt_criteria =    '44120' '44121' '44122' '44123' '44124' '44125' '44126' '44127' '44128' '44129' '44130' '44131' '44132' '44133' '44134' '44135' '44136' '44137' '44138' '44139' '44140' '44141' '44142' '44143' '44144' '44145' '44146' '44147' '44148' '44149' '44150' '44151' '44152' '44153' '44154' '44155' '44156' '44157' '44158' '44159' '44160' '44202' '44203' '44204' '44205' '44206' '44207' '44208' '44209' '44210' '44211' '44212' '44213' '44227' '44625' '44626' '45100' '45101' '45102' '45103' '45104' '45105' '45106' '45107' '45108' '45109' '45110' '45111' '45112' '45113' '45114' '45115' '45116' '45117' '45118' '45119' '45120' '45121' '45122' '45123' '45124' '45125' '45126' '45127' '45128' '45129' '45130' '45131' '45132' '45133' '45134' '45135''45136' '45137' '45138' '45139' '45140' '45141' '45142' '45143' '45144' '45145' '45146' '45147' '45148' '45149''45150' '45151' '45152' '45153' '45154' '45155' '45156' '45157' '45158' '45159' '45160' '45161' '45162' '45163' '45164 '45165' '45166' '45167' '45168' '45169' '45170' '45171' '45172'  '45395' '45397' '46020' '46030' '46040' '46045' '46050' '46060' '46258' '46270' '46275' '46280' '46285' '46288' '46706' '46707' '46715' '57300' '57305' '57307' '57308';   \| Source \| Code \| \| --- \| --- \| \| ICD-9 \| 173x Laparoscopic multiple segmental resection of large intestine \| \| ICD-9 \| 456x Other Excision Of Small Intestine \| \| ICD-9 \| 457x Open And Other Partial Excision Of Large Intestine \| \| ICD-9 \| 458x Total Intra-Abdominal Colectomy \| \| ICD-9 \| 459x Intestinal Anastomosis \| \| ICD-9 \| 46xx Other Operations On Intestine \| \| ICD-9 \| 484x Pull-Through Resection Of Rectum \| \| ICD-9 \| 485x Abdominoperineal Resection Of Rectum \| \| ICD-9 \| 486x Other Resection Of Rectum \| \| ICD-9 \| 491x Incision Or Excision Of Anal Fistula \| \| ICD-9 \| 4973 Closure Of Anal Fistula \| \| ICD-9 \| 5783 Repair Of Fistula Involving Bladder And Intestine \| \| ICD-9 \| 7072 Repair Of Colovaginal Fistula \| \| ICD-9 \| 7073 Repair Of Rectovaginal Fistula \| \| ICD-9 \| 7074 Repair Of Other Vaginoenteric Fistula \| \| ICD-10 \| 0D Gastrointestinal System \|      \| CPT Code \| Description \| \| --- \| --- \| \| 44120 \| Enterectomy, resection of small intestine; single resection and anastomosis \| \| 44125 \| Enterectomy, resection of small intestine; with enterostomy \| \| 44126 \| Enterectomy, resection of small intestine for congenital atresia, single resection and anastomosis of proximal segment of intestine; without tapering \| \| 44127 \| Enterectomy, resection of small intestine for congenital atresia, single resection and anastomosis of proximal segment of intestine; with tapering \| \| 44130 \| Enteroenterostomy, anastomosis of intestine, with or without cutaneous enterostomy (separate procedure) \| \| 44137 \| Removal of transplanted intestinal allograft, complete \| \| 44140 \| Colectomy, partial; with anastomosis \| \| 44141 \| Colectomy, partial; with skin level cecostomy or colostomy \| \| 44143 \| Colectomy, partial; with end colostomy and closure of distal segment (Hartmann type procedure) \| \| 44144 \| Colectomy, partial; with resection, with colostomy or ileostomy and creation of mucofistula \| \| 44145 \| Colectomy, partial; with coloproctostomy (low pelvic anastomosis) \| \| 44146 \| Colectomy, partial; with coloproctostomy (low pelvic anastomosis), with colostomy \| \| 44147 \| Colectomy, partial; abdominal and transanal approach \| \| 44150 \| Colectomy, total, abdominal, without proctectomy; with ileostomy or ileoproctostomy \| \| 44151 \| Colectomy, total, abdominal, without proctectomy; with continent ileostomy \| \| 44155 \| Colectomy, total, abdominal, with proctectomy; with ileostomy \| \| 44156 \| Colectomy, total, abdominal, with proctectomy; with continent ileostomy \| \| 44157 \| Colectomy, total, abdominal, with proctectomy; with ileoanal anastomosis, includes loop ileostomy, and rectal mucosectomy, when performed \| \| 44158 \| Colectomy, total, abdominal, with proctectomy; with ileoanal anastomosis, creation of ileal reservoir (S or J), includes loop ileostomy, and rectal mucosectomy, when performed \| \| 44160 \| Colectomy, partial, with removal of terminal ileum with ileocolostomy \| \| 44202 \| Laparoscopy, surgical; enterectomy, resection of small intestine, single resection and anastomosis \| \| 44204 \| Laparoscopy, surgical; colectomy, partial, with anastomosis \| \| 44205 \| Laparoscopy, surgical; colectomy, partial, with removal of terminal ileum with ileocolostomy \| \| 44206 \| Laparoscopy, surgical; colectomy, partial, with end colostomy and closure of distal segment (Hartmann type procedure) \| \| 44207 \| Laparoscopy, surgical; colectomy, partial, with anastomosis, with coloproctostomy (low pelvic anastomosis) \| \| 44208 \| Laparoscopy, surgical; colectomy, partial, with anastomosis, with coloproctostomy (low pelvic anastomosis) with colostomy \| \| 44210 \| Laparoscopy, surgical; colectomy, total, abdominal, without proctectomy, with ileostomy or ileoproctostomy \| \| 44211 \| Laparoscopy, surgical; colectomy, total, abdominal, with proctectomy, with ileoanal anastomosis, creation of ileal reservoir (S or J), with loop ileostomy, includes rectal mucosectomy, when performed \| \| 44212 \| Laparoscopy, surgical; colectomy, total, abdominal, with proctectomy, with ileostomy \| \| 44213 \| Laparoscopy, surgical, mobilization (take-down) of splenic flexure performed in conjunction with partial colectomy (List separately in addition to primary procedure) \| \| 44227 \| Laparoscopy, surgical, closure of enterostomy, large or small intestine, with resection and anastomosis \| \| 44625 \| Closure of enterostomy, large or small intestine; with resection and anastomosis other than colorectal \| \| 44626 \| Closure of enterostomy, large or small intestine; with resection and colorectal anastomosis (eg, closure of Hartmann type procedure) \| \| 45110 \| Proctectomy; complete, combined abdominoperineal, with colostomy \| \| 45111 \| Proctectomy; partial resection of rectum, transabdominal approach \| \| 45112 \| Proctectomy, combined abdominoperineal, pull-through procedure (eg, colo-anal anastomosis) \| \| 45113 \| Proctectomy, partial, with rectal mucosectomy, ileoanal anastomosis, creation of ileal reservoir (S or J), with or without loop ileostomy \| \| 45114 \| Proctectomy, partial, with anastomosis; abdominal and transsacral approach \| \| 45116 \| Proctectomy, partial, with anastomosis; transsacral approach only (Kraske type) \| \| 45119 \| Proctectomy, combined abdominoperineal pull-through procedure (eg, colo-anal anastomosis), with creation of colonic reservoir (eg, J-pouch), with diverting enterostomy when performed \| \| 45120 \| Proctectomy, complete (for congenital megacolon), abdominal and perineal approach; with pull-through procedure and anastomosis (eg, Swenson, Duhamel, or Soave type operation) \| \| 45121 \| Proctectomy, complete (for congenital megacolon), abdominal and perineal approach; with subtotal or total colectomy, with multiple biopsies \| \| 45126 \| Pelvic exenteration for colorectal malignancy, with proctectomy (with or without colostomy), with removal of bladder and ureteral transplantations, and/or hysterectomy, or cervicectomy, with or without removal of tube(s), with or without removal of ovary(s), or any combination thereof \| \| 45136 \| Excision of ileoanal reservoir with ileostomy \| \| 45395 \| Laparoscopy, surgical; proctectomy, complete, combined abdominoperineal, with colostomy \| \| 45397 \| Laparoscopy, surgical; proctectomy, combined abdominoperineal pull-through procedure (eg, colo-anal anastomosis), with creation of colonic reservoir (eg, J-pouch), with diverting enterostomy, when performed \| \| 57307 \| Closure of rectovaginal fistula; abdominal approach, with concomitant colostomy \| |

Supplemental Table 2. Demographics of the Medicaid Population with 1+ Crohn’s Disease Diagnosis Code Stratified by Sex, 2010-2019

|  |  | Never Used Tobacco | | Used Tobacco After Dx | | Used Tobacco Before Dx | | Ever Used Tobacco | |
| --- | --- | --- | --- | --- | --- | --- | --- | --- | --- |
|  |  | Female | Male | Female | Male | Female | Male | Female | Male |
| Age at 1^st^ Medicaid Eligibility (%) | 0-5 | 0.47 | 0.99 | 0 | 0 | 0 | 0 | 0 | 0 |
|  | 6-10 | 3.47 | 7.56 | 0.00 | 0.00 | 0.00 | 0.00 | 0.07 | 0.00 |
|  | 11-20 | 12.49 | 19.55 | 6.22 | 8.39 | 4.64 | 4.89 | 5.37 | 6.16 |
|  | 21-30 | 14.61 | 13.51 | 22.99 | 17.21 | 20.46 | 16.02 | 20.68 | 16.37 |
|  | 31-40 | 15.60 | 14.07 | 24.72 | 20.02 | 22.12 | 19.27 | 22.52 | 19.09 |
|  | 41-50 | 16.57 | 16.00 | 24.05 | 26.52 | 24.17 | 26.82 | 23.70 | 26.26 |
|  | 51-60 | 16.27 | 15.27 | 16.16 | 21.33 | 19.05 | 22.49 | 18.55 | 22.14 |
|  | 61-70 | 11.61 | 8.29 | 4.84 | 4.99 | 7.16 | 7.83 | 6.95 | 7.39 |
|  | 71-105 | 8.91 | 4.76 | 0.93 | 1.39 | 2.35 | 2.57 | 2.16 | 2.48 |
| Race (%) | White, non-Hispanic | 51.36 | 51.43 | 72.50 | 63.78 | 68.97 | 60.89 | 69.28 | 60.70 |
|  | Black, non-Hispanic | 15.15 | 13.32 | 15.42 | 15.37 | 16.43 | 16.76 | 16.41 | 16.33 |
|  | Other/unknown | 33.49 | 35.25 | 12.08 | 20.85 | 14.60 | 22.35 | 14.31 | 22.97 |
| 1^st^ Year of Medicaid Eligibility (%) | 2010 | 62.10 | 57.86 | 89.83 | 84.83 | 78.63 | 69.29 | 80.80 | 72.27 |
|  | 2011 | 5.01 | 5.08 | 6.10 | 8.14 | 6.77 | 8.19 | 6.43 | 7.86 |
|  | 2012 | 3.15 | 3.21 | 2.19 | 3.80 | 3.14 | 4.60 | 2.94 | 4.37 |
|  | 2013 | 2.25 | 2.58 | 0.84 | 1.16 | 1.73 | 2.49 | 1.61 | 2.26 |
|  | 2014 | 6.31 | 8.40 | 1.03 | 2.07 | 4.14 | 7.89 | 3.60 | 6.92 |
|  | 2015 | 4.00 | 4.81 | 0.00 | 0.00 | 1.08 | 1.94 | 0.89 | 1.62 |
|  | 2016 | 15.92 | 16.27 | 0.00 | 0.00 | 4.50 | 5.59 | 3.72 | 4.68 |
|  | 2017 | 1.27 | 1.79 | 0.00 | 0.00 | 0.00 | 0.00 | 0.00 | 0.00 |
| 1^st^ Year of Eligible CD Encounter (%) | 2011 | 17.63 | 15.52 | 38.95 | 35.03 | 22.56 | 19.20 | 27.02 | 22.86 |
|  | 2012 | 16.06 | 14.43 | 30.71 | 29.38 | 22.84 | 19.79 | 23.88 | 21.22 |
|  | 2013 | 12.05 | 11.34 | 18.12 | 20.11 | 15.73 | 15.54 | 15.55 | 15.82 |
|  | 2014 | 7.69 | 7.59 | 8.11 | 9.87 | 9.20 | 9.59 | 8.55 | 9.45 |
|  | 2015 | 7.80 | 8.68 | 4.12 | 5.62 | 8.65 | 10.94 | 7.63 | 9.76 |
|  | 2016 | 8.26 | 9.46 | 0.00 | 0.00 | 6.74 | 7.63 | 5.56 | 6.39 |
|  | 2017 | 16.40 | 17.35 | 0.00 | 0.00 | 8.01 | 9.76 | 6.62 | 8.17 |
|  | 2018 | 14.11 | 15.62 | 0.00 | 0.00 | 6.27 | 7.56 | 5.18 | 6.33 |
| Age at 1^st^ CD Encounter (%) | 11-20 | 13.68 | 25.00 | 4.04 | 6.64 | 2.16 | 3.10 | 2.93 | 4.42 |
|  | 21-30 | 13.61 | 13.36 | 20.09 | 14.49 | 16.28 | 12.24 | 16.94 | 12.73 |
|  | 31-40 | 15.42 | 13.88 | 25.74 | 20.53 | 23.08 | 19.49 | 23.43 | 19.52 |
|  | 41-50 | 15.63 | 14.77 | 23.22 | 24.19 | 22.71 | 22.79 | 22.47 | 22.58 |
|  | 51-60 | 18.17 | 17.23 | 19.81 | 26.12 | 23.26 | 28.38 | 22.35 | 27.62 |
|  | 61-70 | 12.20 | 9.49 | 5.64 | 6.18 | 8.98 | 10.33 | 8.64 | 9.64 |
|  | 71-105 | 11.29 | 6.27 | 1.45 | 1.84 | 3.54 | 3.65 | 3.23 | 3.48 |
| Place of Service at 1st CD Encounter | Office | 32.66 | 33.19 | 25.33 | 30.26 | 21.28 | 24.45 | 23.12 | 27.05 |
|  | Inpatient Hospital | 20.74 | 20.09 | 24.22 | 22.69 | 32.23 | 32.41 | 29.25 | 29.06 |
|  | Outpatient Hospital | 18.74 | 18.80 | 27.19 | 25.01 | 21.98 | 19.89 | 23.12 | 20.75 |
|  | Emergency Room - Hospital | 7.56 | 6.91 | 11.80 | 11.15 | 11.75 | 10.18 | 11.43 | 10.05 |
|  | Other | 20.29 | 21.00 | 11.46 | 10.89 | 12.76 | 13.08 | 13.09 | 13.10 |
| 1+ CD Code (%) |  | 100.00 | 100.00 | 100.00 | 100.00 | 100.00 | 100.00 | 100.00 | 100.00 |
| 2+ CD Codes (%) |  | 50.59 | 53.56 | 60.63 | 56.61 | 54.66 | 50.88 | 55.96 | 51.93 |
| Tobacco (%) |  | 0.00 | 0.00 | 100.00 | 100.00 | 100.00 | 100.00 | 100.00 | 100.00 |
| Steroid (%) |  | 55.66 | 51.81 | 69.57 | 59.95 | 64.95 | 53.88 | 65.04 | 54.66 |
| Antibiotic (%) |  | 51.68 | 48.05 | 67.48 | 57.63 | 62.43 | 52.17 | 62.65 | 52.97 |
| 5-ASA (%) |  | 15.62 | 19.75 | 16.12 | 16.11 | 13.97 | 13.67 | 14.30 | 14.59 |
| Immunomodulator (%) |  | 8.14 | 11.44 | 6.94 | 6.21 | 6.05 | 5.40 | 6.31 | 5.81 |
| 6-Mercaptopurine (%) |  | 2.80 | 4.13 | 2.11 | 2.55 | 1.67 | 2.06 | 1.81 | 2.31 |
| Azathioprine (%) |  | 3.51 | 3.80 | 3.22 | 2.33 | 2.87 | 2.37 | 2.96 | 2.41 |
| Methotrexate (%) |  | 2.51 | 4.47 | 2.17 | 1.59 | 1.99 | 1.32 | 2.02 | 1.42 |
| Biologic (%) |  | 10.67 | 15.07 | 9.08 | 8.71 | 8.21 | 7.63 | 8.53 | 8.10 |
| Anti-TNF (%) |  | 9.72 | 14.09 | 8.80 | 8.56 | 7.83 | 7.20 | 8.15 | 7.73 |
| Adalimumab (%) |  | 5.32 | 7.04 | 5.64 | 4.57 | 5.04 | 3.84 | 5.16 | 4.10 |
| Certolizumab Pegol (%) |  | 0.54 | 0.43 | 0.79 | 0.71 | 0.59 | 0.39 | 0.66 | 0.45 |
| Golimumab (%) |  | 0.15 | 0.13 | 0.00 | 0.00 | 0.10 | 0.00 | 0.11 | 0.00 |
| Infliximab (%) |  | 5.31 | 8.24 | 4.15 | 4.65 | 3.60 | 3.97 | 3.82 | 4.24 |
| JAK Inhibitor (%) |  | 0.16 | 0.17 | 0.00 | 0.00 | 0.08 | 0.00 | 0.07 | 0.00 |
| Tofacitinib (%) |  | 0.16 | 0.17 | 0.00 | 0.00 | 0.08 | 0.00 | 0.07 | 0.00 |
| Upadacitinib (%) |  | 0.00 | 0.00 | 0.00 | 0.00 | 0.00 | 0.00 | 0.00 | 0.00 |
| Natalizumab (%) |  | 0.04 | 0.00 | 0.00 | 0.00 | 0.00 | 0.00 | 0.00 | 0.00 |
| Ustekinumab (%) |  | 0.86 | 0.98 | 0.45 | 0.40 | 0.54 | 0.45 | 0.52 | 0.40 |
| Vedolizumab (%) |  | 1.27 | 1.39 | 0.71 | 0.48 | 0.72 | 0.68 | 0.74 | 0.63 |
| Cyclosporine (%) |  | 0.00 | 0.00 | 0.00 | 0.00 | 0.00 | 0.00 | 0.00 | 0.00 |
| TPN (%) |  | 6.59 | 7.19 | 6.53 | 5.59 | 9.03 | 8.62 | 8.33 | 7.85 |
| Fistula (%) |  | 3.54 | 5.55 | 4.76 | 5.70 | 4.54 | 6.12 | 4.41 | 5.93 |
| Intestinal Fistula (%) |  | 1.72 | 2.36 | 2.57 | 2.92 | 2.56 | 3.35 | 2.40 | 3.19 |
| Perianal Fistula (%) |  | 1.74 | 3.74 | 2.29 | 3.57 | 2.02 | 3.75 | 2.03 | 3.65 |
| Rectovaginal Fistula (%) |  | 0.75 | 0.00 | 1.24 | 0.00 | 1.11 | 0.00 | 1.09 | 0.00 |
| IBD Hospitalization (%) |  | 24.75 | 24.00 | 33.80 | 31.57 | 38.60 | 36.56 | 35.80 | 33.73 |
| IBD Surgery (%) |  | 12.08 | 14.87 | 13.19 | 16.53 | 15.37 | 18.32 | 14.39 | 17.57 |

Supplemental Table 3a. Odds Ratios for Effect of Tobacco Use After Crohn’s Disease Diagnosis on Outcomes in Medicaid Population Stratified by Sex, 2010-2019

|  | Female | | | Male | | |
| --- | --- | --- | --- | --- | --- | --- |
|  | Odds Ratio | Lower CL | Upper CL | Odds Ratio | Lower CL | Upper CL |
| Anti-TNF | 0.915 | 0.835 | 1.003 | 0.882 | 0.771 | 1.009 |
| Biologic | 0.879 | 0.803 | 0.962 | 0.825 | 0.723 | 0.942 |
| Fistula | 1.103 | 0.974 | 1.249 | 0.911 | 0.775 | 1.072 |
| IBD Hospitalization | 0.990 | 0.936 | 1.047 | 0.963 | 0.886 | 1.046 |
| IBD Surgery | 0.905 | 0.839 | 0.977 | 0.974 | 0.880 | 1.078 |
| Steroid | 1.430 | 1.352 | 1.513 | 1.449 | 1.341 | 1.566 |

Supplemental Table 3b. Odds Ratios for Effect of Tobacco Use Before Crohn’s Disease Diagnosis on Outcomes in Medicaid Population Stratified by Sex, 2010-2019

|  | Female | | | Male | | |
| --- | --- | --- | --- | --- | --- | --- |
|  | Odds Ratio | Lower CL | Upper CL | Odds Ratio | Lower CL | Upper CL |
| Anti-TNF | 0.858 | 0.793 | 0.928 | 0.724 | 0.650 | 0.806 |
| Biologic | 0.823 | 0.763 | 0.888 | 0.708 | 0.638 | 0.787 |
| Fistula | 1.137 | 1.024 | 1.263 | 1.170 | 1.035 | 1.323 |
| IBD Hospitalization | 1.859 | 1.777 | 1.945 | 1.829 | 1.719 | 1.946 |
| IBD Surgery | 1.392 | 1.310 | 1.478 | 1.298 | 1.203 | 1.400 |
| Steroid | 1.267 | 1.211 | 1.325 | 1.163 | 1.096 | 1.234 |
